# The Measurement of Stiffness for Major Muscles with Shear Wave Elastography and Myoton: A Quantitative Analysis Study

**DOI:** 10.3390/diagnostics11030524

**Published:** 2021-03-15

**Authors:** Youngjin Lee, Minkyoung Kim, Haneul Lee

**Affiliations:** 1 Department of Radiology Science, Gachon University, Incheon 21936, Korea; yj20@gachon.ac.kr (Y.L.); kkokko5858@naver.com (M.K.); 2 Department of Radiology, Kwanghye Hospital, Seoul 06174, Korea; 3 Department of Physical Therapy, Gachon University, Incheon 21936, Korea

**Keywords:** muscle, myotonometer, quantitative analysis, ultrasound, shear-wave elastography

## Abstract

The present study was performed to assess the relationship between hand-held myotonometer MyotonPRO and shear wave elastography (SWE) measurements of lower limb muscle stiffness during resting and active voluntary contraction. Forty healthy young adults, (20 males and 20 females) participated in the study. The stiffness of each subject’s rectus femoris (RF), biceps femoris (BF), tibialis anterior (TA), and medial gastrocnemius (MG) was measured repeatedly by MyotonPRO and SWE. Moderate to strong correlations between the two methods’ measurements were found for both resting and active voluntary contraction. (*r* = 0.416–0.669, *p* < 0.05; *r* = 0.398–0.594, *p* < 0.05, respectively). Muscle stiffness at rest was significantly lower compared contraction in all four muscles measured by both methods (*p* < 0.05). Intra-rater reliabilities were generally lower when measurements were taken during contraction. Additionally, when compared by gender, muscle stiffness measured by MyotonPRO was significantly higher at rest in men compared to women, except for the TA. However, a significant difference was found in TA muscle stiffness by gender when measured with SWE. When muscles were contracted, all muscles showed significantly higher stiffness in men compared to women. There were moderate to good correlations in muscle stiffness between measurements of SWE and MyotonPRO at rest and during active voluntary contraction. Additionally, both instruments showed good intra-rater reliability.

## 1. Introduction

For the movement of body segments, skeletal muscles are the active and primary driver [1,2]. Soft tissues such as muscles, tendons, aponeuroses, and ligaments are responsible for rapid force production. For improving sports performance and preventing injuries, it is helpful to understand the mechanical properties of these tissues [3]. Structural changes to these tissues alter their elasticity, which leads to a greater risk of musculoskeletal injuries during activities [4]. Measurements of tissue stiffness can be useful for detecting muscle stiffness and many disorders of the organs [5]. Hence, assessing the mechanical properties of tissues is important for health professionals.

In evaluating the mechanical properties of the biological tissues, dynamic elastography methods based on ultrasound imaging modalities are being developed [6]. The elastography methods are largely classified into two types, strain and shear wave. These were recently found to assess the mechanical properties of tissues by calculating the propagation velocity of shear waves using imaging techniques [7]. The first method developed and used for elastography was the strain method, which uses the principle of applying external pressure to the tissue [8]. In addition, this method displays the relative strain and generates a quantitative stiffness map using the relative distortion image [8]. However, elastography using strain has the major disadvantages that the object being observed can be moved out of focus by pressure and that tissue far from the skin surface is difficult to observe [8]. To cope with these disadvantages, shear wave elastography (SWE) was introduced and developed [9]. SWE is a new dynamic tool recommended for assessing the elasticity of tissues by recognizing tissue deformities arising after movements of compression and relaxation [9]. SWE is based on an ultrafast sequence process and non-invasive technique that can detect the elasticity of various tissues [9,10,11,12]. Due to its high-resolution images, it is helpful for examining the mechanical properties of the superficial and deep tissues. SWE’s use in biomechanical research has increased with efficient implications in physical training and rehabilitation [13,14]. It is a quantitative method of assessment of tissue stiffness which helps to achieve rather a direct measure of tissue stiffness, unlike strain elastography [8]. SWE was found to autogenerate and trace the transient shear waves propagating in the tissues [10,15,16]. It calculates the shear wave speed propagation $\left(v_{s}\right)$ within the tissues [15]. SWE works by determining the shear modulus $\left(G\right)$ in the region of interest (ROI) with the combination of an ultrafast acquisition imaging system and radiation force [1].

Recently, SWE has become known to be more useful for evaluating the mechanical properties of tendons, along with those of muscles and peripheral nerves [17]. MyotonPRO, a non-invasive and portable hand-held device, produces valid and genuine results for superficial skeletal muscle palpation [12]. It is a device equipped for muscle stiffness measurements [18]. It may stand as the primary assessment tool for measuring muscle stiffness.

The purpose of this study was to assess the relationship between the measurements of lower limb muscle stiffness by the hand-held myotonometer myotonPRO and by SWE, at rest and during voluntary contraction.

## 2. Materials and Methods

### 2.1. Ethical Approval

This study was approved by the Institutional Review Board of Gachon University (1044396-201908-HR-139-01 and 21 October 2019). All participants gave their written informed consent prior to participation in the study. This cross-sectional study is in accordance with the Strengthening the Reporting of Observational Studies in Epidemiology (STROBE) statement.

### 2.2. Participants

A total of 40 healthy young adults, including 20 males and 20 females, consented to participate in this study. All the participants were healthy—having a body mass index (BMI) between 18 and 25 kg/m^2^ and participating in >150 min of intense physical activity per week. Participants were excluded if they had orthopedic, musculoskeletal, or neurological limitations, or pain in the lower limbs at the time. The demographics of the participants are displayed in Table 1.

### 2.3. Experimental Protocol

Upon arriving at the laboratory, the participants rested comfortably in an ambient-temperature room for 10 min to stabilize them. General characteristics (height, weight, and BMI) were measured before beginning the test. All the participants were educated in how to contract each muscle and asked to practice active voluntary contraction for familiarization. Another 10 min was given to the participants to get relaxed.

The participants were instructed to relax each target muscle for 30 s while MyotonPRO and SWE stiffness were obtained. Then, they contracted each target muscle as instructed and maintained contraction for 10 s for MyotonPRO measurement and another 10 s for SWE measurement; 30 s was given to relax the muscle between the two methods. Muscles included were following; rectus femoris (RF), biceps femoris (BF), tibialis anterior (TA), and medial gastrocnemius (MG).

### 2.4. Measurement Position

The skin over the muscle belly areas was exposed for assessment., Assessment points were marked above the largest cross-section of each muscle belly and length was measured from anatomical landmarks of the dominant leg so that identical points were measured on repeat measures for MyotonPRO and SWE [19].

*Rectus Femoris:* The stiffness of resting RF was measured in supine position. Active voluntary contraction was done at 90° knee flexion in a short seat—we asked the participants to perform 15° hip flexion with their arms across the chest. The measurements were done at 1/3 of the distance between the anterior superior iliac spine and patella.

*Biceps Femoris:* For measuring the stiffness of the BF, participants were asked to lie prone for both resting and contraction. The active voluntary contraction of the BF was done by flexion of the knee joint till 45°. The measurement point was marked at 1/4 of the distance between posterior superior iliac spine and the tendon of the BF.

*Tibialis Anterior:* The participants were asked to lie in a supine position for the measurement of the resting stiffness of the TA. The stiffness of the contracted TA was also measured in supine position with 15° dorsiflexion of the foot.

*Medial Gastrocnemius:* The participants were placed in the prone position with their feet off the edge of the bed. The measurement point was marked at the largest cross-section of the muscle belly. For measuring the stiffness of the contracted MG, the participants were asked to perform 35° plantar flexion.

### 2.5. Quantitative Evaluation Method

**Figure 1:**
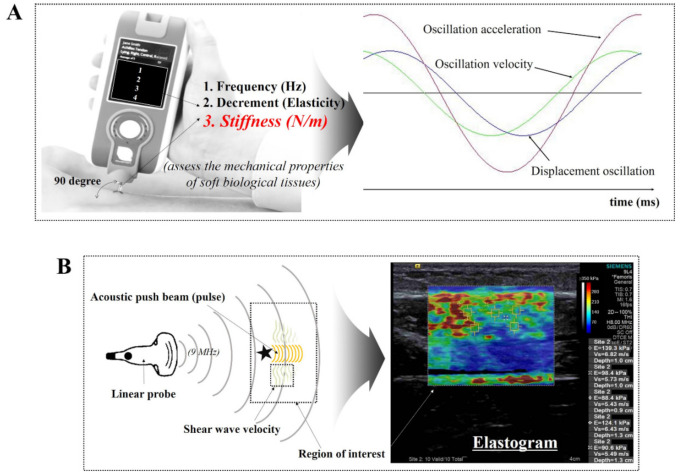
Schematic diagram of (**A**) MyotonPRO, including the measurement method for oscillation performance with respect to time [25], and (**B**) shear wave elastography using a linear probe and acoustic push pulse.

Figure 1 illustrates the schematic diagrams of MyotonPRO, including the measurement method for oscillation performance with respect to time, and SWE, using a linear probe and acoustic push pulse.

SWE uses the principle of obtaining the elasticity by the horizontal generation of shear waves in the direction of the travelling sound wave. This elastography measures the elasticity of a target area based on the principle of using detection pulses sent out from multiple channels after generating a shear wave at the site by concentrating high-intensity ultrasound at a certain area in the tissue [20]. In this study, an SWE (ACUSON S3000, Siemens Healthcare, Erlangen, Germany) ultrasound imaging device with a 9 MHz linear array probe was used. Our linear probe consisted of a high-density element array with a fine pitch (more elements in a given area). In particular, our SWE can acquire high resolution using a wide frequency bandwidth and narrow slice thickness. For quantitative evaluation of stiffness, participants were positioned in various way at rest and during contraction. Before measuring, the stiffness quality factor (QF), which had to be 55 on-screen or more, was evaluated in all cases. After a satisfactory QF result, the averages for stiffness using shear wave speed propagation and pressure with 5 regions of interests (ROIs) in each image were evaluated.

A hand-held digital palpation device, MyotonPRO (Myoton AS, Tallinn, Estonia), was used to measure the superficial skeletal muscle stiffness of the lower limb. This reliable and valid device has been used to measure non-vital physiological parameters of superficial, soft biological tissue both clinically and in research [12,21,22,23] The standard 3 mm diameter-probe was placed perpendicularly to the skin’s surface, directly above the muscle. An initial force of 0.18 N was exerted; then, an additional mechanical force of 0.4 N was applied to the subcutaneous tissue for 15 milliseconds, which induced muscle deformation. Then, the resultant damped natural oscillations caused by the viscoelastic properties of the soft tissue were recorded using a built-in accelerometer at a sampling rate of 3200 Hz [24]. Dynamic stiffness (N/m) of muscle tissue is calculated as a ratio *a*_max_ *m*/Δ*l* with a pre-compression of 0.18 N. The maximum acceleration of the damped oscillation *a*_max_ characterizes the resistance to an external force that deforms the initial shape of the tissue and Δ*l* is described as at the point of maximum displacement of muscle tissue [24].

### 2.6. Reliability Tests

The intra-operator reliability values for muscle stiffness were gathered in 5-randomly selected participants. The general characteristics of 5 participants for the reliability test were not significantly different from the rest of the participants in the study (*p* > 0.05). All measurements were repeated two times in the same scanning session.

### 2.7. Statistical Analysis

SPSS 25.0 software (IBM, Armonk, NY, USA) was used to analyze the data, which were summarized as means and standard deviations (SD) for quantitative variables. The assumption of normality of the continuous variables was examined using the Kolmogorov–Smirnov test. Pearson’s correlation analysis was used to examine the relationship between muscle stiffness measured by MyotonPRO and SWE at each measurement site. An independent *t* test was conducted to compare mean muscle stiffness and elasticity between males and females at each measurement site and in each position. The level of significance was set at α = 0.05.

## 3. Results

### 3.1. Relationship between MyotonPRO and SWE for Measurement of Muscle Stiffness at Rest and during Contraction

**Figure 2:**
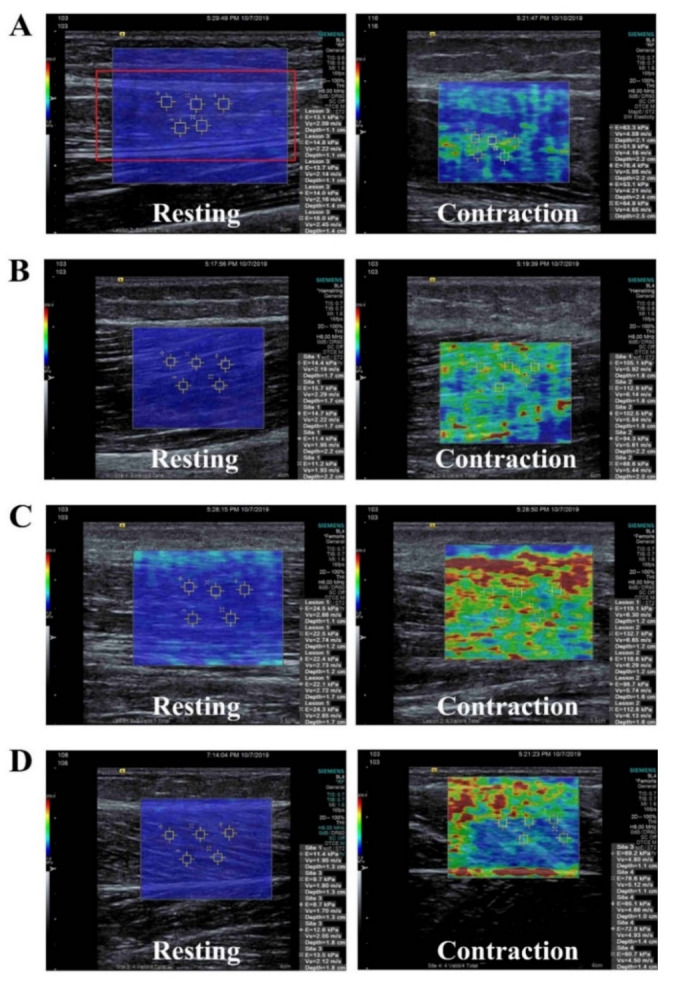
Shear-wave elastography images for measurements of (**A**) rectus femoris, (**B**) biceps femoris, (**C**) tibialis anterior, and (**D**) medial gastrocnemius at rest and during active voluntary contraction.

Table 2 describes muscle stiffness at rest and during contraction measured by MyotonPRO and SWE. Moderate to strong correlations were found between the values of all muscles at rest determined with MyotonPRO and SWE. Similar correlations were found when the muscles were contracted (Table 2). Muscle stiffness at rest was significantly lower compared to stiffness during contraction in all four muscles measured by the MyotonPRO. Muscle stiffness obtained with SWE also showed significantly higher stiffness when muscle was voluntarily contracted than when at rest (Figure 2).

### 3.2. The Difference in Muscle Stiffness between Men and Women

The muscle stiffness of the RF, BF, and MG measured by MyotonPRO was significantly higher at resting position in men compared to women (*p* < 0.05), but no statistically difference was found for the TA. However, a significant difference was found for the TA stiffness by gender when measured with SWE (*p* < 0.01). When muscles were contracted, all four muscles showed significantly higher stiffness in men compared to women when determined by MyotonPRO or SWE (Table 3).

### 3.3. Intra-Rater Reliability of MyotonPro and SWE when Measuring Muscle Stiffness

The intra-rater reliabilities for measurements of all four muscles measured with MyotonPRO and SWE were both good to excellent at rest and during contraction. The intra-rater reliability values for interclass correlation coefficient (ICC) are presented in Table 4. Intra-rater reliabilities were lower when measurements were taken during contraction than when taken at rest.

## 4. Discussion

The current study examined the stiffness of four muscles, including the RF, TA, BF, and MG, using the hand-held myotonometer MyotonPRO and SWE when muscles were at rest and when they were contracting to verify the correspondence between the two measurement methods. Our results showed a moderate-to-strong positive correlation in muscle stiffness detection between MyotonPRO and SWE. The relationship was stronger for resting measurements (r = 0.416–0.669) compared to those during contraction (r = 0.398–0.594) for all four muscles.

In the present study, intra-rater reliabilities of measuring muscle stiffness were slightly higher when using MyotonPRO compared to SWE. Intra-rater reliabilities of measuring stiffness by SWE were even lower when the muscle was actively contracted compared to resting. That might have been due to the vague guidelines of talking ROIs. Although SWE can obtain high-quality visuals with ultrasound images, there is a major disadvantage in that each result can differ depending on the ROI set by operator. Thus, an image-based color mapping program is needed to acquire high reliability regardless of the ROI setting.

In comparison with SWE, MyotonPRO showed quite consistent results, which might have been due to the measurement probe being pointy (small). It, however, could be a disadvantage of the MyotonPRO because the measurable area is limited.

Nevertheless, our results indicate the MyotonPRO is a reliable method with which to examine superficial muscle stiffness. The results also support the previous studies. Feng et al. showed good intra-operator repeatability of MyotonPRO compared with SWE in the measurement of the stiffness of the gastrocnemius muscle and Achilles tendon, but the study only measured stiffness at rest for the muscle [12]. Similarly, but in a more specific manner, Kelley et al. showed a medium to good correlation when examining muscle stiffness using MyotonPro and SWE at various contraction levels [4]. They also reported that the greater the muscle contraction intensity applied, the higher the muscle stiffness detected, but measurements obtained by SWE were less precise when the muscle was contracted [4], and our results support the previous research as well. Compared to Kelley et al., our study focused more on muscles of the lower limbs. Additionally, the present research compared the muscle stiffness between genders with two different measurement methods to see if the results were consistent. The muscle stiffness values of the RF, BF, and MG measured by MyotonPRO were significantly greater in men compared to women at rest, except TA, but with SWE even TA stiffness was significantly different by gender. When muscles were contracted, all four muscles showed significantly greater stiffness in men compared to women when determined by MyotonPRO or SWE. Muscles consist of collagen fibers closely packed together. Due to the decrease of collagen formation and fibroblast proliferation with increased estrogen, the decreased collagen synthesis causes weak muscle strength and elasticity [26]. The effects of sex hormones on the structure and mechanical properties of human connective tissue have been studied, and there is an obvious sex-related difference in the role of estrogen in regulating muscle mass [27,28]. A recent study examined muscle stiffness measured by MyotonPRO and found a significantly lower stiffness of the TA and peroneus longus during ovulation compared to during the follicular phase [29], which relates to the results in the current study.

Even though we assessed the mechanical properties of several muscles in the lower limbs at rest and during active voluntary contraction and compared them by gender, the present study has several limitations need to be addressed. Firstly, active voluntary contraction was not measured by quantitatively using electromyography or a dynamometer, but by specific joint angles. Secondly, subcutaneous fat was not measured when muscle stiffness was measured by MyotonPro. Previous evidence states that MyotonPRO is not suitable for measuring muscle stiffness when muscles are covered with subcutaneous fat of more than 20 mm. Further studies should measure subcutaneous fat to control that covariate. Lastly, this study included only healthy young adults, so further research should include different types of subjects, such as healthy elderly people or stroke patients to compare and generalize the results for each population.

SWE uses the basic principle of analyzing the waveform of the wave spreading in the vertical direction after compressing the tissue using an ultrasound probe [20], while MyotonPRO basically uses the method of calculating the natural frequency of the body’s skin and the physiological properties of the muscle [30]. Although the quantitative values of muscle stiffness derived through/from the two instruments are different, a consistent tendency and high correlation have been demonstrated in our study.

In summary, our results have shown a moderate to good correlation in muscle stiffness between measurements by SWE and MyotonPRO at rest and during active voluntary contraction. Additionally, both instruments showed good intra-rater reliability. This study could be a starting point for studies examining these variables in various populations with soft tissue or neuromuscular disorders.

## Figures and Tables

**Table 1:** General characteristics of participants.

	Total (*n* = 40)	Men (*n* = 20)	Women (*n* = 20)
Age (years)	22.15 ± 2.29	22.75 ± 2.9	21.6 ± 1.28
Height (Cm)	168.10 ± 8.58	174.35 ± 5.91	161.85 ± 5.82
Weight (kg)	63.53 ± 12.53	70.45 ± 12.61	56.62 ± 7.90
BMI (kg/m^2^)	22.32 ± 2.88	23.06 ± 3.15	21.57 ± 2.44

Abbreviation: BMI—body mass index.

**Table 2:** Correlation coefficients of muscle stiffness values for MyotonPRO and shear wave elastography (SWE) at rest and during active voluntary contraction (*N* = 40).

Muscle		MyotonPRO (N/m) (Mean ± SD)	SWE (kPa) (Mean ± SD)	*r*
Rectus Femoris	R	245.15 ± 36.84	13.99 ± 3.14	0.416 **
	C	366.43 ± 88.94	79.76 ± 14.99	0.398 *
Tibialis Anterior	R	365.74 ± 35.67	21.10 ± 2.99	0.561 **
	C	814.18 ± 170.58	138.97 ± 32.80	0.540 **
Biceps Femoris	R	263.63 ± 57.00	13.13 ± 3.70	0.652 **
	C	369.12 ± 144.80	80.45 ± 15.74	0.594 **
Medial Gastrocnemius	R	278.25 ± 31.67	12.29 ± 2.84	0.669 **
	C	381.55 ± 117.47	79.68 ± 18.45	0.551 **

Abbreviation: R, resting; C, contraction. ** Correlation is significant at the 0.01 level. * Correlation is significant at the 0.05 level.

**Table 3:** Differences in muscle stiffness between men and women (*N* = 40).

Muscle		MyotonPRO (N/m) (Mean ± SD)		SWE (kPa) (Mean ± SD)	
		Men	Women	Men	Women
Rectus Femoris	R	269.20 ± 28.79	221.10 ± 27.20 **	15.47 ± 2.68	12.51 ± 2.91 **
	C	441.80 ± 112.77	291.05 ± 66.05 **	84.66 ± 13.93	74.85 ± 14.71 *
Tibialis Anterior	R	374.50 ± 37.53	356.53 ± 32.02	22.27 ± 2.58	19.86 ± 2.95 **
	C	920.85 ± 138.99	701.89 ± 122.83 **	150.19 ± 28.35	127.15 ± 26.48 *
Biceps Femoris	R	307.55 ± 38.88	219.7 ± 33.09 **	15.72 ± 2.86	10.54 ± 2.42 **
	C	479.26 ± 124.89	258.98 ± 43.62 **	88.31 ± 12.86	72.17 ± 14.40 **
Medial Gastrocnemius	R	269.90 ± 25.24	226.60 ± 20.86 **	13.85 ± 2.72	10.73 ± 2.00 **
	C	456.69 ± 116.27	314.32 ± 68.66 **	83.72 ± 20.74	65.83 ± 15.54 *

Abbreviation: R, resting; C, contraction. ** Significant difference between men and women at the 0.01 level. * Significant difference between men and women at the 0.05 level.

**Table 4:** Intra-rater reliability of MyotonPRO and SWE in measurements of muscle stiffness.

Muscle		MyotonPRO		SWE	
		ICC	95% CI	ICC	95% CI
Rectus Femoris	R	0.938	0.153, 0.989	0.916	0.196, 0.994
	C	0.872	0.036, 0.998	0.790	−0.277, 0.998
Tibialis Anterior	R	0.880	0.006, 0.992	0.852	−0.171, 0.992
	C	0.894	0.074, 0.993	0.814	−0.073, 0.948
Biceps Femoris	R	0.884	−0.381, 0.981	0.842	−0.140, 0.989
	C	0.861	−0.073, 0.990	0.715	−0.439, 0.979
Medial Gastrocnemius	R	0.904	0.122, 0.993	0.876	−0.011, 0.991
	C	0.856	−0.091, 0.990	0.763	−0.365, 0.990

Abbreviation: R, resting; C, contraction; CI, confidence interval; ICC, interclass correlation coefficient.

## Data Availability

The datasets generated during the current study are available from the corresponding author on reasonable request.
